# The Activity of Antimicrobial Surfaces Varies by Testing Protocol Utilized

**DOI:** 10.1371/journal.pone.0160728

**Published:** 2016-08-05

**Authors:** Matias D. Campos, Paola C. Zucchi, Ann Phung, Steven N. Leonard, Elizabeth B. Hirsch

**Affiliations:** Department of Pharmacy and Health Systems Sciences, Northeastern University, Boston, Massachusetts, United States of America; Cleveland Clinic, UNITED STATES

## Abstract

**Background:**

Contaminated hospital surfaces are an important source of nosocomial infections. A major obstacle in marketing antimicrobial surfaces is a lack of efficacy data based on standardized testing protocols.

**Aim:**

We compared the efficacy of multiple testing protocols against several “antimicrobial” film surfaces.

**Methods:**

Four clinical isolates were used: one *Escherichia coli*, one *Klebsiella pneumoniae*, and two *Staphylococcus aureus* strains. Two industry methods (modified ISO 22196 and ASTM E2149), a “dried droplet”, and a “transfer” method were tested against two commercially available antimicrobial films, one film in development, an untreated control, and a positive (silver) control film. At 2 (only ISO) and 24 hours following inoculation, bacteria were collected from film surfaces and enumerated.

**Results:**

Compared to untreated films in all protocols, there were no significant differences in recovery on either commercial brand at 2 or 24 hours after inoculation. The silver surface demonstrated significant microbicidal activity (mean loss 4.9 Log_10_ CFU/ml) in all methods and time points with the exception of 2 hours in the ISO protocol and the transfer method. Using our novel droplet method, no differences between placebo and active surfaces were detected. The surface in development demonstrated variable activity depending on method, organism, and time point. The ISO demonstrated minimal activity at 2 hours but significant activity at 24 hours (mean 4.5 Log_10_ CFU/ml difference versus placebo). The ASTEM protocol exhibited significant differences in recovery of staphylococci (mean 5 Log_10_ CFU/ml) but not Gram-negative isolates (10 fold decrease). Minimal activity was observed with this film in the transfer method.

**Conclusions:**

Varying results between protocols suggested that efficacy of antimicrobial surfaces cannot be easily and reproducibly compared. Clinical use should be considered and further development of representative methods is needed.

## Introduction

Contamination of hospital surfaces with nosocomial pathogens has long been recognized as an important source of infection. In response, extensive guidelines on control and prevention of contamination are in place, created by equipment manufacturers, public health departments, and healthcare organizations [1–3]. This environment has been acknowledged an important “reservoir” for microorganisms responsible for hospital-acquired infections [4]. It has been documented that hospital patients often shed organisms into their environments which can be picked up by healthcare workers [1, 5–9]. Furthermore, certain organisms can persist on surfaces for up to 4–5 months or more at concentrations sufficient for transmission [1].

In an attempt to reduce rates of nosocomial infection, there is heightened interest in developing surfaces with antimicrobial properties. At the present time, stainless steel is common in hospital settings due to appearance, durability, and ease of cleaning. However, this material does not have inherent antimicrobial properties and studies have demonstrated that *Clostridium difficile* spores and methicillin-resistant *Staphylococcus aureus* (MRSA) can survive on stainless steel for up to five and seven months or more, respectively [4]. However, metallic copper surfaces have antimicrobial properties, demonstrating significant activity within 2 hours of inoculation [10]. Use of copper alloy surfaces on highly touched surfaces in intensive care units has translated into clinical efficacy by reducing relative risk of hospital-acquired infections by more than 50% [11]. Another study utilizing copper oxide-impregnated textiles reduced hospital-acquired infection per 1000-hospitalization days by 24% in a long-term care ward [12].

A major obstacle in marketing novel antimicrobial surfaces is a lack of performance evidence based on efficacy test protocols. The testing protocol commonly used in industry is International Standard (ISO) 22196 titled “Plastics–Measurement of antibacterial activity on plastics surfaces [4, 13].” In this protocol, surfaces are tested using a liquid bacterial culture, covered in a plastic film, and grown for 24 hours at high temperature and 100% relative humidity. Bacteria are recovered and counted at time of 0 hours and 24 hours. This test is widely used by manufacturers because it optimizes the chance for the antibacterial component of the surface to contact the liquid culture to confirm activity before commercialization [13]. However, Ojeil et al have described this method as inappropriate to test antimicrobial surfaces due to artificial experimental conditions including high temperatures of 37°C, high 100% relative humidity, and a direct liquid presentation of the bacterial culture [4]. Another industry standard is the American Society of the International Association for Testing and Materials (ASTM) E2149 protocol titled “Standard Test Method for Determining the Antimicrobial Activity of Antimicrobial Agents Under Dynamic Contact Conditions [14].” In this test, test surfaces are agitated in inoculum for the duration of the experiment at 37°C. Neither of these testing conditions reflect the conditions found in practice^.^ Consequently, the results of an ISO 22196 or ASTM E31249 experiment do not generally reflect the actual activity of these surfaces in the clinical setting.

Therefore, in an effort to better simulate conditions found in practice and to optimally characterize the activity of antimicrobial surface products, we compared multiple protocols using nosocomial pathogens. ASTM E2149, another industry standard, a modified ISO 22196, and two novel testing protocols were used in order to better simulate commercial conditions of these products.

## Methods

### Test film surfaces

Three antimicrobial films were tested along with a positive and negative control film. Two test films were commercially available antimicrobial iPad screen protectors manufactured by OnGuard (Antimicrobial Screen Protector for 3^rd^/4^th^ Generation iPads, Waltham, MA) and Boxwave (ClearTouch Anti-Microbial, Kirkland, WA), while the third test film was under development (‘film in development’; FID). Both OnGuard and Boxwave films utilized silver particles as the active component [15, 16]. The active ingredients in the FID are modified nano-inorganic particles (personal communication). They function by promoting and maintaining the production of radicals, which are involved in bacterial killing. The positive control film was a silver film (Kodak X-Omat, Carestream Health, Inc. Rochester, NY) and the negative control film was an inactive version of the FID without the active ingredient. Best attempts were made to maintain the silver surface in the dark, in storage and during testing. This was in an effort to limit silver oxidation reactions to light. Film surfaces measured 1 inch x 2 inches and were not treated upon receipt. Four different methods were used to determine the killing activity of each of the films as described below.

### Bacterial strains

Four clinical bacterial isolates were used in the experiments including two Gram-negative isolates collected from CHI St. Luke’s in Houston, TX (*Escherichia coli* 9927 [CTX-M-1 extended-spectrum beta-lactamase (ESBL)-producing] and *Klebsiella pneumoniae* 9936 [CTX-M-1 and SHV ESBL-producing]) and two *Staphylococcus aureus* strains collected from Brigham and Women’s Hospital in Boston, MA (*Staphyloccocus aureus* 95 [methicillin-susceptible, MSSA] and *Staphylococcus aureus* 175 [methicillin-resistant]). All strains were stored at -80°C on CryoCare beads (Key Scientific Products, Stamford, TX) and subcultured twice on blood agar plates prior to experiments.

### International Organization for Standardization (ISO) 22196: Measurement of antibacterial activity on plastics and other non-porous surfaces

A modified version of ISO 22196 was followed [13]. This is currently the test protocol of choice for testing surfaces with an antimicrobial claim [4]. Ten mL of Mueller-Hinton broth (MHB; Becton, Dickinson and Company. Sparks, MD) was inoculated and incubated overnight in a shaker at 37°C. On the day of testing, 200 μL of overnight culture was grown in 10 mL of fresh MHB, shaking at 37°C for 1 hour to achieve exponential-phase growth. Using a spectrophotometer, this 1-hour culture was further diluted in saline to reach a cell density of 7 Log_10_ colony forming units (CFU)/mL. Each film surface was then inoculated with 50 μL of the 7 Log_10_ CFU/mL (to achieve a final density of 5.7 Log_10_ CFU/mL on the film) culture and covered with a plastic film to ensure maximum contact time and to prevent drying. Films were incubated at room temperature in a small plastic box containing a piece of paper saturated with water to maintain high humidity. Bacterial burden at 2 h and 24 h was determined by transferring films to a 50 mL conical tube filled with 10 mL 0.9% sodium chloride solution and agitating for 30 seconds to recover inoculum. One mL of resulting solution was serially diluted, plated on MHA, and incubated for 24 h at 37°C to determine the resulting CFU. Log_10_ reductions were calculated by subtracting time point log_10_ count on control and test surfaces from the initial inoculum.

### ASTM E2149: Standard Test Method for Determining the Antimicrobial Activity of Antimicrobial Agents under Dynamic Contact Conditions

Ten mL of tryptic soy broth (TSB) was inoculated and incubated overnight in a shaker at 37°C [14]. On the day of testing, this culture was diluted using 0.3 mM KH_2_PO_4_ buffer solution to a concentration of 8.3 Log_10_ CFU/mL, verified using a spectrophotometer. Several 250 mL Erlenmeyer flasks were prepared with 25 mL of KH_2_PO_4_ buffer, one for each strain and surface including an “inoculum only” flask. The 8.3 Log_10_ CFU/mL culture was further diluted in these flasks to a final concentration of 5.3 Log_10_ CFU/mL. One mL of the “inoculum only” flask was serially diluted, plated on MHA, and enumerated to verify the starting inoculum. Each flask received a respective film piece and was placed in a shaker bath for 24 hours at 37°C. After 24 hours, 1 mL of solution from each flask was serially diluted, plated on MHA, and enumerated. Log_10_ reductions were calculated by subtracting time point log_10_ count on control and test surfaces from the initial inoculum.

### Novel dried droplet method

This method was developed in our lab in an effort to simulate a more clinically relevant/realistic environment of dried droplets following coughing, sneezing, or release of respiratory secretions. Cultures were prepared using two methods: from both a 1-hour log-phase growth flask and from a colony selected from an overnight culture on a blood agar plate. For 1-hour growth, ten mL of MHB was inoculated and incubated overnight in a shaker at 37°C. On the day of testing, overnight culture was grown in fresh MHB to achieve exponential-phase growth. Using a spectrophotometer, this culture was further diluted in saline to reach an 8 Log_10_ CFU/mL concentration. For the overnight cultures, several loops of culture were inoculated into 10 mL of MHB to a concentration of log 8 CFU/mL, verified using a spectrophotometer. Four 1.5 μL drops of the resulting log 8 CFU/mL culture were placed on each surface and incubated at room temperature and room humidity for 24 hours. Bacteria were collected with a cotton swab moistened with 100 μL of saline and diluted into test tubes containing 900 μL saline. These tubes were agitated for 1 minute and the resulting solution was serially diluted, plated, and incubated to determine bacterial counts. Log_10_ reductions were calculated by subtracting time point log_10_ count on control and test surfaces from the initial inoculum.

### Transfer method

For this protocol, only the FID and placebo films were compared. Overnight cultures were grown at 37°C in cation-adjusted MHB in a water shaker bath. On the day of testing, this culture was added to artificial saliva (mucin 1.35g/L; KCl 475mg/L; NaCl 700 mg/L; CaCl [dehydrate] 185 mg/L; K_2_HPO_4_ 105 mg/L; MgCl_2_∙6H2O 105 mg/L; urea 45 mg/L; D(+)glucose 100 mg/L; alpha-amylase 100,000 U/L) to achieve a suspension of approximately 8 Log_10_ CFU/mL. Twenty-five μL of this suspension was inoculated onto two 1” x 1” glass slides and placed into a controlled environment chamber (model 5518; Electro-tech Systems, Inc. Glenside, PA) to dry at 65% relative humidity and room temperature. Immediately after drying, one set of slides (the “donor” slides) was sonicated in 50 mL conical tubes containing 20 mL of saline. Using the second set of slides, each strain was transferred to a placebo film and a FID test surface by placing the film in contact with the slide and applying pressure with a roller and rolling approximately 20 times. The transferred films (both test and placebo) were incubated under desired conditions for 2 or 24 hrs. Bacteria were then recovered from the film surfaces by sonicating the films in conical tubes containing 20 mL of saline. After sonication, the resulting solution was serially diluted and bacterial counts were enumerated on MHA after incubation.

## Results

### ISO 22196 protocol

**Fig 1:**
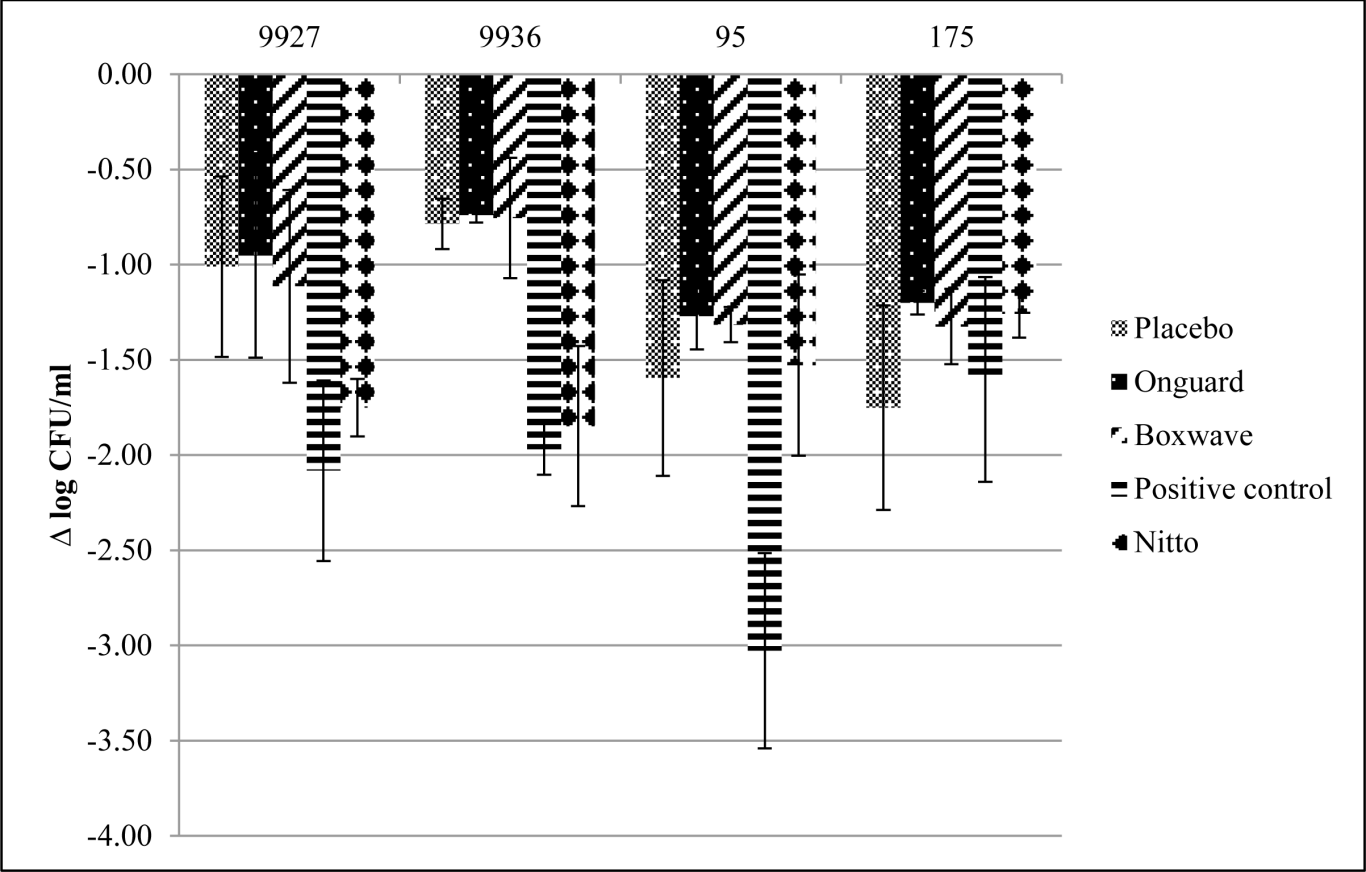
Mean ± standard deviation Log_10_ reduction from initial inocula after 2 h using ISO 22196.

**Fig 2:**
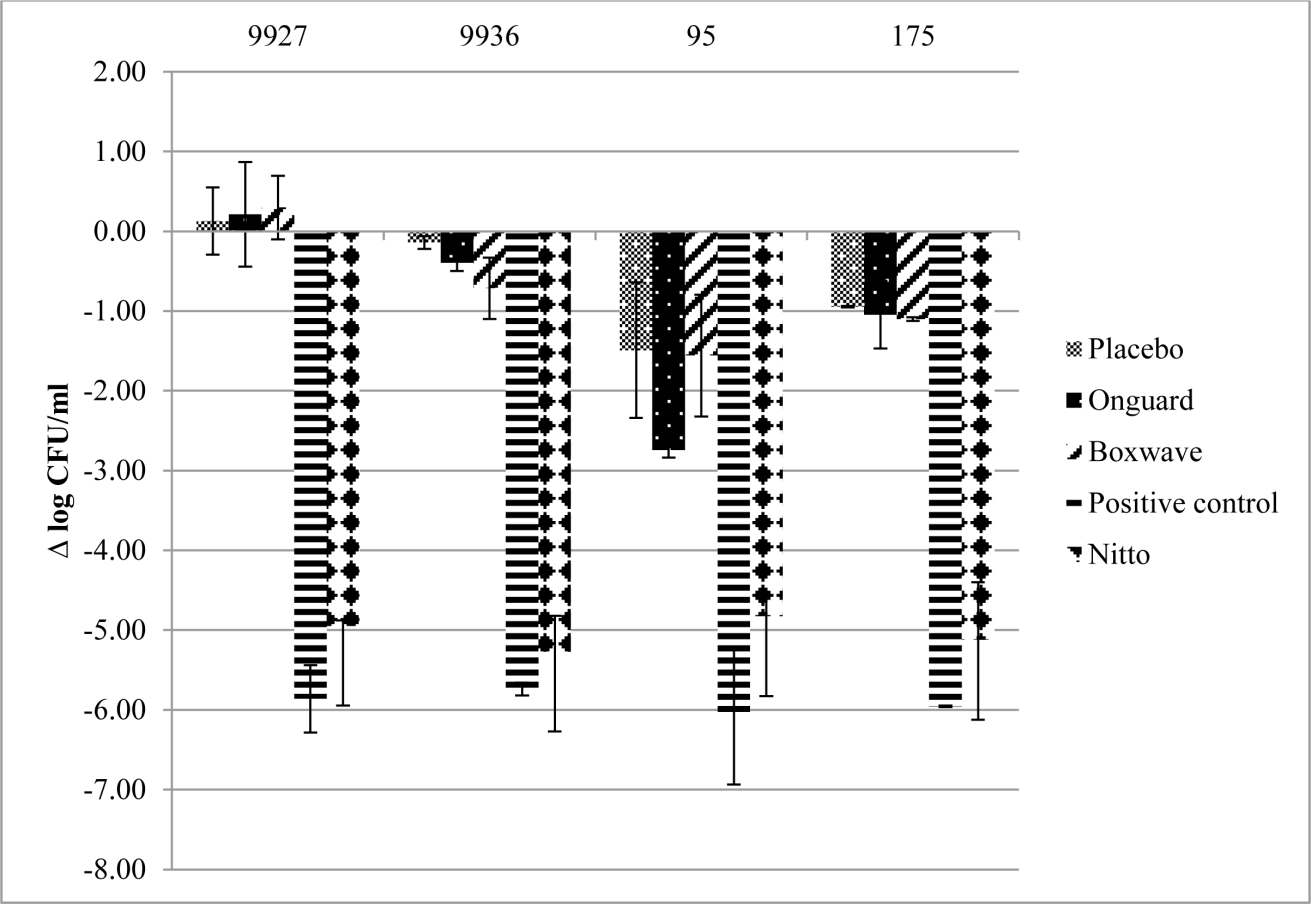
Mean ± standard deviation Log_10_ reduction from initial inocula after 24 h using ISO 22196.

The antibacterial activity of the test surfaces against the four strains using ISO 22196 after 2 and 24 hours is presented in Figs 1 and 2, respectively. The mean initial inoculum on these surfaces was 5.90 ± 0.4 log_10_ CFU/mL. At 2 h and 24 h, the commercially available films exhibited minimal to no activity compared to placebo. At 2 h, the FID and positive control demonstrated variable activity, depending on the strain tested. The FID surface exhibited more activity against the Gram-negative species, 9927 and 9936, and was similar to the positive control film. The positive control (silver) surface was more active against all strains, except MRSA 195. However, at 24 hours, both the FID and silver surface showed >4 log_10_ CFU/mL reductions in viable bacteria.

### ASTM E2149 protocol

**Fig 3:**
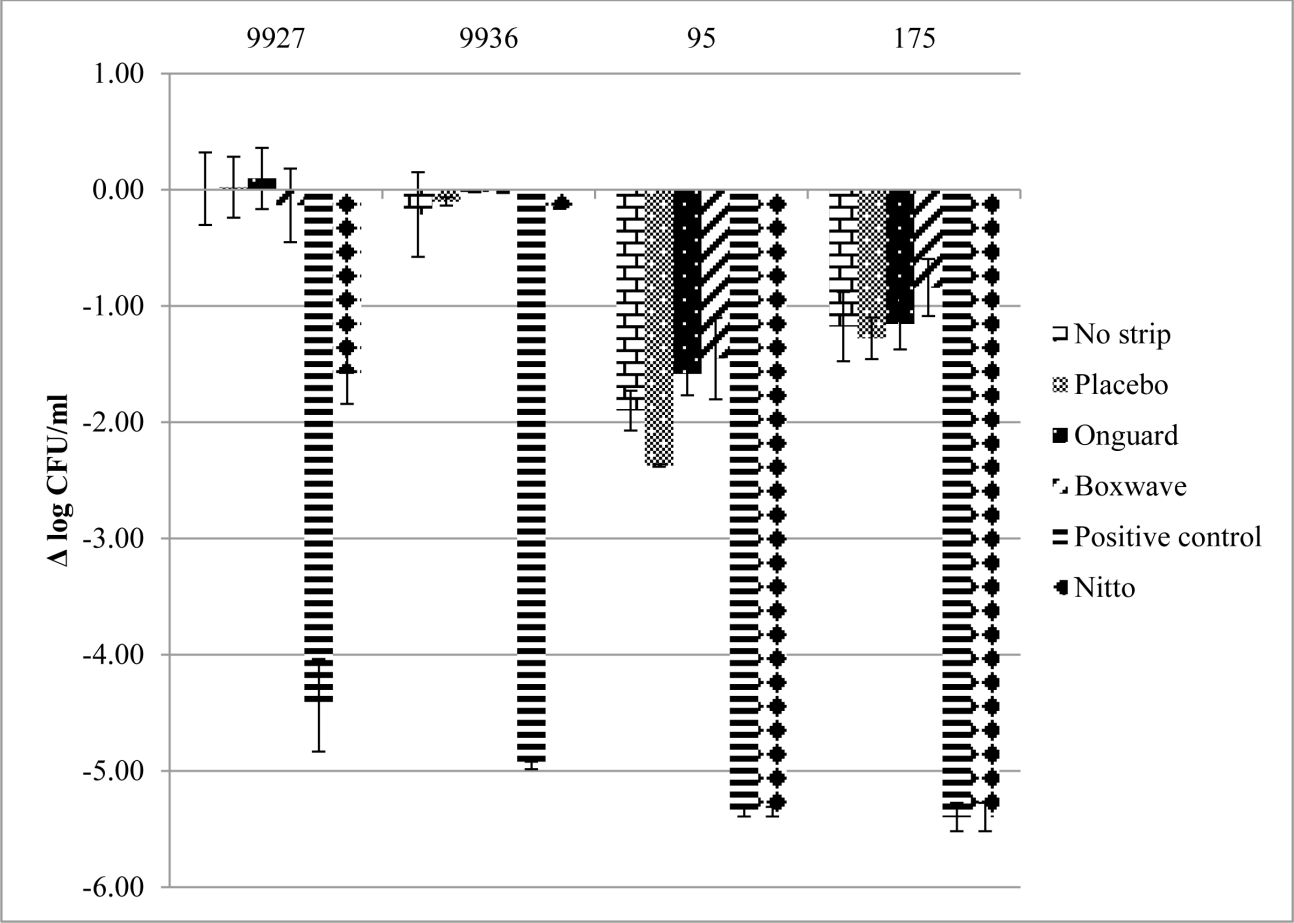
Mean ± standard deviation Log_10_ reduction from initial inocula after 24 h using ASTM E2149.

The efficacy of test surfaces using the ASTM E2149 method is presented in Fig 3. The mean initial inoculum on these surfaces was 5.53 ± 0.19 log_10_ CFU/mL. Minimal antimicrobial activity was seen using the commercially available films when compared to placebo for this method. Significant antibactericidal activity was seen with the FID surface with >4 log_10_ reduction against *S*. *aureus* but with minimal activity (mean reduction 0.87 ± 0.83 log_10_ CFU/mL) against the Gram-negative species. However, the positive control demonstrated significant activity against all strains with >4 log_10_ reductions in viable bacteria.

### Dried droplet protocol

The antibacterial activity of the test surfaces against the four strains using the dried droplet protocol (from 1-hr log-phase growth) after 24 hours is presented in Table 1. No activity was observed with the commercially available brands when compared to placebo. In this method, variable but minimal activity in the positive control and FID films was seen. The positive control demonstrated some activity against most strains, except 9927. The FID surface demonstrated somewhat less activity against all strains. When compared to the bacterial colony inoculum preparation (data not shown), minimal deviations were seen from the broth preparation.

**Table 1:** Mean ± standard deviation Log_10_ reduction (CFU/mL) from initial inocula after 24 h using the dried droplet protocol.

Isolate	Film type				
	Placebo	Onguard	Boxwave	Positive control	FID
9927	-4.44 ± 1.83	-4.82 ± 0.77	-4.68 ± 1.02	-4.35	-5.15 ± 0.85
9936	-3.88 ± 1.71	-4.34 ± 0.96	-4.20 ± 0.82	-6.59	-5.05 ± 1.59
95	-3.70 ± 2.30	-3.73 ± 0.04	-4.14 ± 0.43	-7.16	-4.76 ± 0.96
175	-3.64 ± 1.35	-3.50 ± 0.43	-3.41 ± 0.38	-4.62	-4.49 ± 1.58

### Transfer method

**Fig 4:**
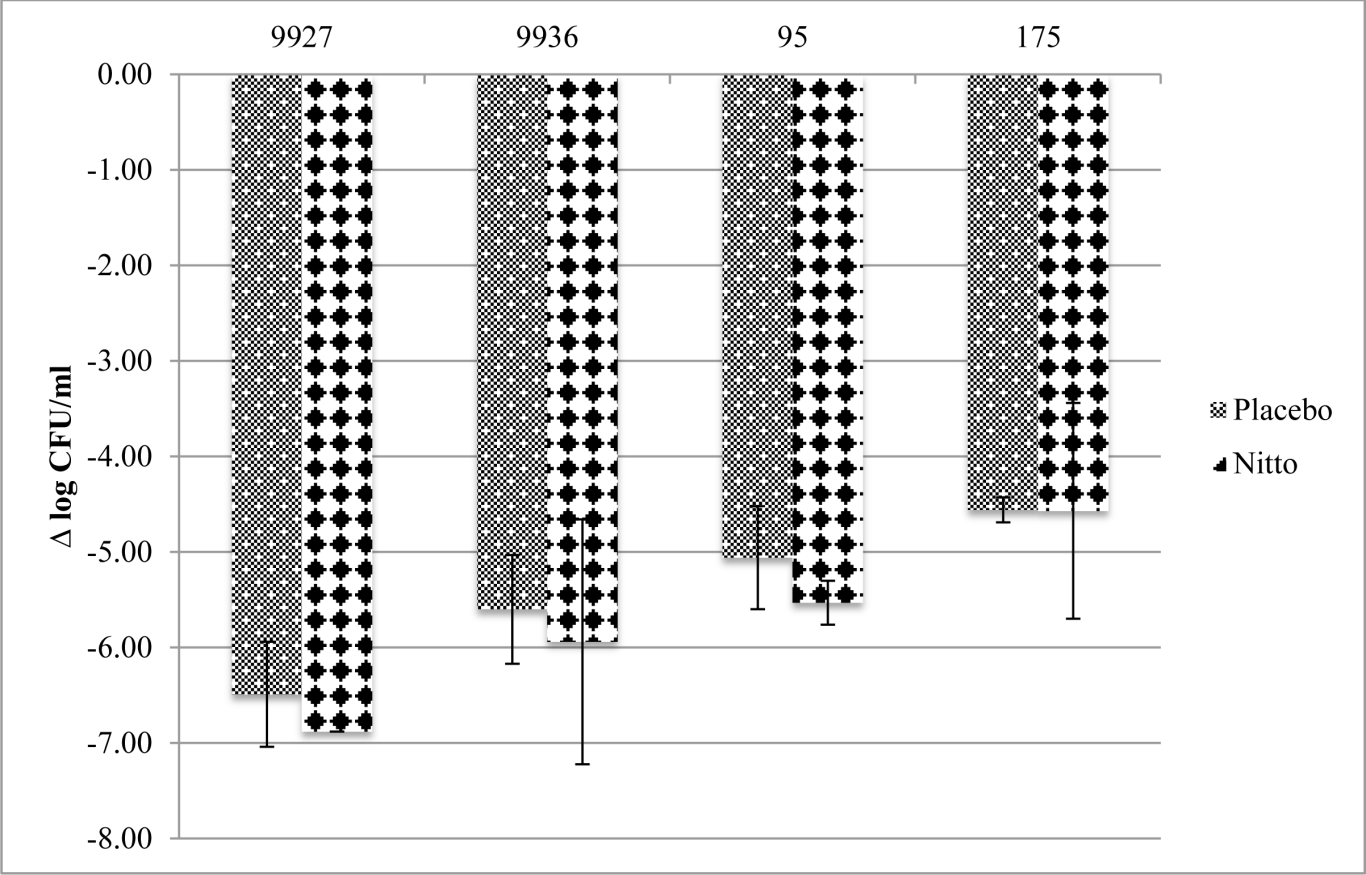
Mean ± standard deviation Log_10_ reduction (CFU/mL) from initial inocula after 2 h using transfer method

**Fig 5:**
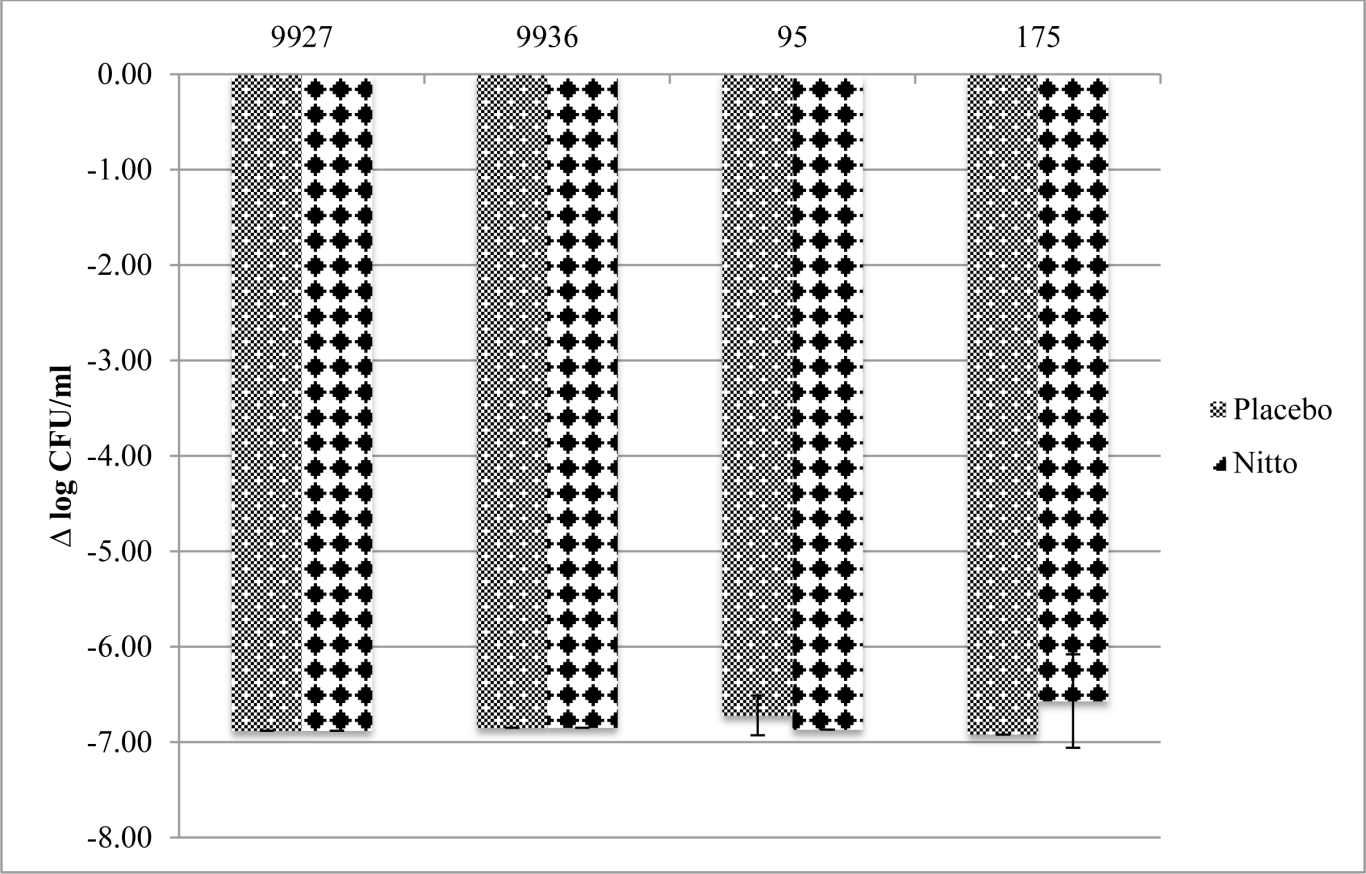
Mean ± standard deviation Log_10_ reduction (CFU/mL) from initial inocula after 24 h using transfer method

The antibacterial activity of the FID test surface against the four strains using the transfer method after 2 and 24 h is presented in Figs 4 and 5, respectively. No overall difference in bacterial recovery/killing of the active film compared to placebo was seen at either the 2 h or 24 h time points. There were modest decreases in the mean Log_10_ CFU/mL change at 2 h for three of the four strains. However, these differences were within acceptable error limits. MSSA 95 seemed to be most susceptible to the active films, with a 3 log_10_ CFU/mL difference against placebo at the 2 h time point.

## Discussion

Hospital surfaces are considered an important fomite for nosocomial pathogens and subsequent infection. These organisms can persist on surfaces for several months at concentrations sufficient for transmission. For example, strains of *Clostridium difficile* spores and methicillin-resistant *Staphylococcus aureus* (MRSA) have demonstrated viability on stainless steel several months after inoculation. In an effort to reduce contamination of these surfaces, extensive guidelines on control and prevention have been implemented. Another option has been development of surfaces with antimicrobial properties. A significant issue with these surfaces has been a lack of reliable, reproducible, and standardized methods for determining antibacterial activity.

Both the ISO 22196 and ASTM E2149 are established, straightforward tests for determining the antimicrobial activity of surfaces that allege antimicrobial activity. These protocols are primarily used in the industry to substantiate marketing claims. The data obtained from the ISO 22196 showed that only the FID film tested had antibacterial activity at 24 h. There was limited or no activity by the other films at both time points and the FID film at 2 h. Results from the ASTM E2149 were comparable to the ISO 22196 for MRSA 95 and MSSA 175 only, with limited activity against the other two strains. Similarly, the other films in the ASTM E2149 saw no activity at 24 h. Both of these tests were conducted at 37°C and 100% relative humidity for the specified duration of the incubation period.

We used an additional two methods, the dried droplet and transfer method, for determining antimicrobial efficacy of surfaces. Compared to placebo controls, the dried droplet method demonstrated very limited antimicrobial activity in the two commercially available films. It was likely desiccation which contributed to reduced bacterial recovery with commercially available films and controls, rather than film activity. As previously described by Ojeil et al [4], reduced efficacy of copper surfaces was seen at lower humidity. Variable but limited activity was seen with the FID film and the silver control film. Minimal deviations were seen among the two inoculate preparations. Similar results were seen in the transfer method. While only the FID film was tested, there was no difference in activity between placebo and FID. These two tests suggested that the active films did not have antimicrobial properties.

There was significant variability in film activity depending on the method utilized to test antimicrobial effect. The two commercially available brands were unable to demonstrate any efficacy, regardless of the method used. The two established methods, ISO 22196 and ASTM E2149, were able to demonstrate some antimicrobial activity with the FID film. There existed some variability of activity, depending on the time point and organism. However, we suggest that ISO 22196 and ASTM E2149 are not appropriate efficacy tests to prove application of surfaces to be used in clinical settings. The main reason for this stems from the high temperature and high relative humidity of testing conditions in these two methods. They are not representative of the true conditions for proposed use in clinical settings. Surfaces may exhibit antimicrobial activity under these extreme conditions but not at a lower temperature and lower relative humidity found in most indoor settings. This could potentially lead to results that seemingly show antimicrobial properties of surfaces or films that may not function in real-world situations. Therefore, two additional methods were developed and tested in an effort to mimic real-life conditions. Sneezing, talking, coughing, and vomiting in clinical environments can create droplets with infectious material [17, 18]. Potential other sources of droplet infection include nebulizers, ventilation and air-conditioning systems [19]. These particles can vary in size from 0.01 to 500 μm and in sick patients from 0.05 to 500 μm [20]. The use of droplets might reflect better the incubation of bacteria on surfaces following these conditions. The dried droplet method utilized four 1.5 μL drops on each test surface that were dried at room temperature and humidity. The transfer method utilized larger drops of 25 μL with bacteria suspended in artificial saliva, dried at room temperature and 65% relative humidity. These incubation conditions attempt to better replicate clinical conditions in which antimicrobial surfaces might be used. This should be contrasted with the more artificial test conditions in the ISO 22196 and ASTM E2149 methods.

Currently, there is limited evidence regarding bacterial droplet deposition on surfaces and the effect of antimicrobial surfaces on bacterial viability. Robine et al previously published a comparison between *Enterococcus faecalis* viability and relative humidity [21]. Bacterial droplets on stainless steel tested lived longer in dry media. Low viability was observed in incubation conditions at 0% and 31% relative humidity. However, strains incubated at 85% relative humidity were completely inactivated after 24 h. Likewise, Ojeil et al were only able to discriminate efficacy between different antimicrobial copper alloy surfaces at lower relative humidity [4]. At 100% relative humidity, all surfaces exhibited maximum antimicrobial activity. These authors also utilized bacterial droplets on test surfaces. Similar bacterial viability was seen in the dried droplet and transfer methods that were conducted at room humidity and 65% relative humidity, respectively. No active film demonstrated antibacterial activity in these conditions. Conversely, ISO 22196 and ASTM E2149 were conducted in high humidity and FID films were able to demonstrate some activity. Additionally, we tested four antibiotic-resistant nosocomial pathogens using four testing protocols. This contrasts with stock/control organisms used in other studies. It should be noted that these protocols have several different variables, in addition to relative humidity, to differentiate between them. Nonetheless, relative humidity remains a seemingly important factor in determining testing conditions.

The variability seen in the antimicrobial efficacy of the films calls into question two established methods for testing surfaces. These two methods, ISO 22916 and ASTM E2149, were able to discriminate the efficacy of the FID film. However, the testing conditions of high temperature of 37°C and 100% relative humidity in these methods are not representative of the actual application of these surfaces. When tested in less extreme conditions by the droplet and transfer methods, the FID films were not able to demonstrate similar antimicrobial seen in the established protocols. These experiments have highlighted the importance of environmental and test conditions when assessing the activity of marketed antimicrobial products. Consequently, assessment techniques of any material must be adapted to its final and important application in clinical settings.
